# Mapping evidence on the burden and distribution of childhood obesity in sub-Saharan Africa: a scoping review protocol

**DOI:** 10.1186/s13643-019-1189-z

**Published:** 2019-11-13

**Authors:** Frederick I. Danquah, Matilda Yeboah, Vitalis Bawontuo, Desmond Kuupiel

**Affiliations:** 1 Faculty of Health and Allied Sciences, Catholic University College of Ghana, Fiapre, Sunyani, Ghana; 2 Research for Sustainable Development Consult, Sunyani, Ghana; 3 Discipline of Public Health Medicine, School of Nursing and Public Health, University of KwaZulu-Natal, Durban, South Africa

**Keywords:** Prevalence, Incidence, Mortality, Distribution, Childhood, Obesity, Risk factors, Sub-Saharan Africa

## Abstract

**Background:**

Obesity in childhood is associated with adverse health outcomes and complications throughout the life-span of a child. Available evidence suggests a dramatic increase in childhood obesity in sub-Saharan Africa (SSA) over the past two decades. The health risks associated with obesity/overweight are particularly problematic in children due to the potential for long-term health concerns. The researchers propose to conduct a systematic scoping review to map evidence on the burden and distribution of childhood obesity in SSA.

**Methods:**

The study will be guided by the scoping review framework proposed by Arksey and O’Malley. A comprehensive literature search will be performed in the following electronic databases: PubMed, Web of Science, African Index Medicus, and CINAHL with full text via EBSCOhost platform. Primary studies both published in peer-reviewed journals and gray literature such as unpublished studies, thesis, and studies in press addressing the research topic will be included. One reviewer will conduct title screening, and the results will be exported to Mendeley Desktop library. Two independent reviewers will perform both abstract and full article screening in parallel as well as data extraction from eligible studies. The Preferred Reporting Items for Systematic Reviews and Meta-analysis: Extension for Scoping Review (PRISMA-ScR) will be utilized to present the study findings of the proposed scoping review. NVivo version 11.0 will be used to extract the relevant outcomes from the included studies, a content thematic analysis performed, and the results reported using a narrative approach. The Mixed Method Quality Appraisal Tool Version 2018 will be used to assess the methodological quality of all included studies.

**Discussion:**

We anticipate that the proposed study will contribute to the existing body of knowledge on childhood obesity, identify gaps in knowledge on the topic, inform future research direction, and provide evidence-based information to strengthen health systems and policies on childhood obesity towards achieving the WHO global target of halting the rise in obesity by 2025.

## Background

Nutritional disorders such as obesity and overweight in childhood and adolescence are associated with adverse health consequences throughout the life-span [1]. The World Health Organization (WHO) defined obesity as abnormal or excessive fat accumulation that presents a risk to health [2]. Obesity results from a complex interplay between genetic, environmental, and socioeconomic factors [3]. Globally, the prevalence of obesity has increased by 27.5% for adults and 47.1% for children [3]. It was estimated that in 2010, overweight/obesity accounted for 3.4 million deaths, 3.9% of years of life lost, and 3.8% of disability-adjusted life-years worldwide [4]. Obesity and overweight were once considered as problems only in high-income countries (HICs) but have now risen dramatically in low- and middle-income countries (LMICs), particularly in urban settings [2] which sub-Saharan Africa (SSA) is no exception. There has been a dramatic increase in obesity in SSA over the past two decades [5]. In 2013, the worldwide prevalence of obesity among children under 5 years was reported to be 42 million of which LMICs accounted for more than 30% higher prevalence than HICs [6]. It is therefore crucial that children and adolescents are thought basic life skills, including proper nutrition and adequate physical activity to prevent obesity-related health challenges during adulthood [7].

The WHO classifies obesity as the fifth leading cause of mortality globally and one of the greatest health challenges and determinants for various chronic disease and psychosocial problems [8]. Muthuri et al. assert that the health risks associated with obesity/overweight are particularly problematic in children due to the potential for long-term health concerns [9]. Even though childhood obesity is difficult to diagnose, the body mass index (BMI) percentile charts for age and sex have been widely accepted as the best tool [10]. Body mass index (BMI) is a simple index of weight for height that is commonly used to classify overweight and obesity. It is defined as a person’s weight in kilograms divided by the square of his height in meters (kg/m^2^) [6]. Researchers have recommended diagnosing a child older than 2 years of age as overweight if the BMI is ≥ 85th percentile but < 95th percentile for age and sex, as obese if the BMI is ≥ 95th percentile, and as extremely obese if the BMI is ≥ 120% of the 95th percentile or ≥ 35 kg/m^2^ [11, 12].

Obesity is an epidemic which requires novel approaches including preventive, therapeutic, and sociocultural changes which are multidisciplinary in nature to address [7]. Although many studies on obesity in children and adolescents may have been conducted globally, our search in PubMed online database using the following keywords: “epidemiology,” “prevalence,” “obesity,” “overweight,” “child,” “adolescent,” “adolescence,” “sub-saharan africa,” and “scoping review” with no limitations found no study mapping the prevalence of obesity in SSA. Therefore, the proposed scoping review will aim to map evidence on the burden and distribution of childhood obesity (children age between 2 and 15 years) in SSA. It is expected that the results of this study will reveal the current prevalence, incidence, and mortality of childhood obesity in SSA as well as risk factors accounting for the development of obesity in children. We also anticipate that the proposed scoping review will reveal gaps in literature for future research to enable policy decisions and strategies to reduce the burden and also prevent childhood obesity in SSA.

## Methods

### Overview

We employed the Preferred Reporting Items for Systematic Reviews and Meta-Analysis for protocols (PRISMA-P) to guide this scoping review protocol write-up (Additional file 1). Scoping reviews are important research tools used to map a range of literature that exists around research topics of interest and help to put research questions in perspective by charting existing research findings and identifying research gaps [13]. A scoping study is also considered a useful approach for determining the need and value of future primary studies or a full systematic review [14]. The authors will base this scoping review on the enhanced Arksey and O’Malley scoping review framework which involves the following: identifying the research question; identifying relevant studies, study selection, and charting the data; and collating, summarizing, and reporting results [14, 15].

### Identifying the research question

This scoping review will utilize an amended Population, Exposure, and Outcome (PEO) framework to determine the eligibility of the primary research question as shown in Table 1. The main review question will be: What is the evidence on the burden and distribution of childhood obesity in SSA?

**Table 1:** PEO framework for defining the eligibility of the studies for the primary research question

P—population	Children (persons aged from 2 to 15 years)
E—exposure	Obesity (children with BMI ≥ 85th percentile)
O—outcome	Prevalence
	Incidence
	Mortality
	Distribution (trends)
	Risks factors of childhood obesity

The sub-questions for the proposed scoping review are as follows:

- What is the evidence on the burden (prevalence, incidence, and mortality) of childhood obesity in SSA?
- What is the distribution of the burden of childhood obesity in SSA?
- What are the risks factors associated with childhood obesity in SSA?

### Identifying relevant studies

The authors will conduct a comprehensive and exhaustive search of multiple bibliographic databases to identify all relevant studies on the burden of childhood obesity in SSA regardless of publication status (published, unpublished, and press). We will conduct a complete search in the following online databases: PubMed, African Index Medicus, and CINAHL with full text via EBSCOhost platform, and Web of Science from 2009 to the search date for recent relevant studies using a combination of keywords in each database. These keywords will include the following: “burden,” “prevalence,” “incidence,” “distribution,” “trend,” “childhood,” “children,” “child,” “pediatric,” “pediatric,” “adolescence,” “adolescent,” “youth,” “obesity,” “obese,” “overweight,” “body mass index,” “bmi,” “risks factors,” “Africa,” and “sub Saharan Africa.” We will also include country names as part of our search strategy. Boolean terms, AND/OR, will be used to separate the keywords. Medical Subject Heading (MeSH) terms will also be included in the search. Language limitations will be removed; however, the study design will be limited to only quantitative studies and humans. The reference list of included studies will also be searched for additional relevant studies using SCOPUS. We will also search for gray literature using Google as well as from websites of international organizations and unpublished literature relevant to answer the research question. Details of each search conducted in the databases will be appropriately documented as follows: search date, search engine, keywords, the number of publications retrieved, and the number of eligible studies. Table 2 illustrates a pilot search strategy in PubMed.

**Table 2:** Pilot search in PubMed electronic database

Date	Database	Keywords	Search results
31 August 2019	PubMed	Line 1. “obesity”[MeSH Terms] OR “obesity”[All Fields] OR Line 2. “obese”[All Fields] OR (“overweight”[MeSH Terms] OR “overweight”[All Fields] OR Line 3. “body mass index”[MeSH Terms] OR “body”[All Fields] OR Line 4. “mass”[All Fields] OR “index”[All Fields] OR “body mass index”[All Fields]) OR (bmi [All Fields] AND Line 5. “child”[MeSH Terms] OR “child”[All Fields] OR Line 6. “children”[All Fields] OR “childhood”[All Fields] OR Line 7. “adolescent”[MeSH Terms] OR “adolescent”[All Fields] OR Line 8. “adolescents”[All Fields] OR “adolescence”[All Fields] OR Line 9. “youth”[All Fields] OR (“pediatrics”[MeSH Terms] OR Line 10. “pediatrics”[All Fields] OR “pediatric”[All Fields] OR “pediatric”[All Fields] OR “pediatrics”[All Fields] AND Line 11. (“africa south of the sahara”[MeSH Terms] OR OR “africa south of the sahara”[All Fields] OR “sub Saharan Africa”[All Fields] OR “sub-Saharan Africa” [All Fields] OR “africa”[MeSH Terms] OR “africa”[All Fields] OR “Angola”[All Fields] OR “Benin”[All Fields] OR “Botswana”[All Fields] OR “Burkina Faso”[All Fields] OR “Burundi”[All Fields] OR “Cameroon”[All Fields] OR “Cape Verde”[All Fields] OR “Central African Republic”[All Fields] OR “Chad”[All Fields] OR “Comoros”[All Fields] OR “Congo”[All Fields] OR “Côte d’Ivoire”[All Fields] OR “Djibouti”[All Fields] OR “Equatorial Guinea”[All Fields] OR “Eritrea”[All Fields] OR “Ethiopia”[All Fields] OR “Gabon”[All Fields] OR “The Gambia”[All Fields] OR “Ghana”[All Fields] OR “Guinea”[All Fields] OR “Guinea-Bissau”[All Fields] OR “Kenya”[All Fields] OR “Lesotho”[All Fields] OR “Liberia”[All Fields] OR “Madagascar”[All Fields] OR “Malawi”[All Fields] OR “Mali”[All Fields] OR “Mauritania”[All Fields] OR “Mauritius”[All Fields] OR “Mozambique”[All Fields] OR “Namibia”[All Fields] OR “Niger”[All Fields] OR “Nigeria”[All Fields] OR “Réunion”[All Fields] OR “Rwanda”[All Fields] OR “Sao Tome and Principe”[All Fields] OR “Senegal”[All Fields] OR “Seychelles”[All Fields] OR “Sierra Leone”[All Fields] OR “Somalia”[All Fields] OR “South Africa”[All Fields] OR “Sudan”[All Fields] OR “Swaziland”[All Fields] OR “Tanzania”[All Fields] OR “Togo”[All Fields] OR “Uganda”[All Fields] OR “Western Sahara”[All Fields] OR “Zambia”[All Fields] OR “Zimbabwe”[All Fields] Line 12. ((“2009/01/01”[PDAT]: “2019/12/31”[PDAT]) AND Line 13. “humans”[MeSH Terms])	139,832

### Eligibility criteria and study selection

To ensure the selection of relevant studies for the proposed scoping review, the study selection will be defined by the eligibility criteria as specified under the inclusion/exclusion criteria.

#### Inclusion criteria

The researchers will include studies that meet the following criteria:

- Evidence of study conducted in sub-Saharan Africa
- Studies conducted from 2009 to 2019
- Studies focusing on obesity
- Studies presenting evidence on children aged from 2 to 15 years
- Studies that present evidence on the burden (prevalence, incidence, mortality) of obesity
- Studies that present evidence on the distribution (trends) of obesity
- Studies that present evidence on risks factors of childhood obesity
- Quantitative studies

#### Exclusion criteria

The exclusion criteria will include the following:

- Studies reporting evidence in countries not included in the WHO Africa Region
- Studies targeting individuals below 2 years and above 15 years
- Qualitative studies
- Other types of reviews
- Studies focusing on other methods of assessing body composition

**Fig. 1:**
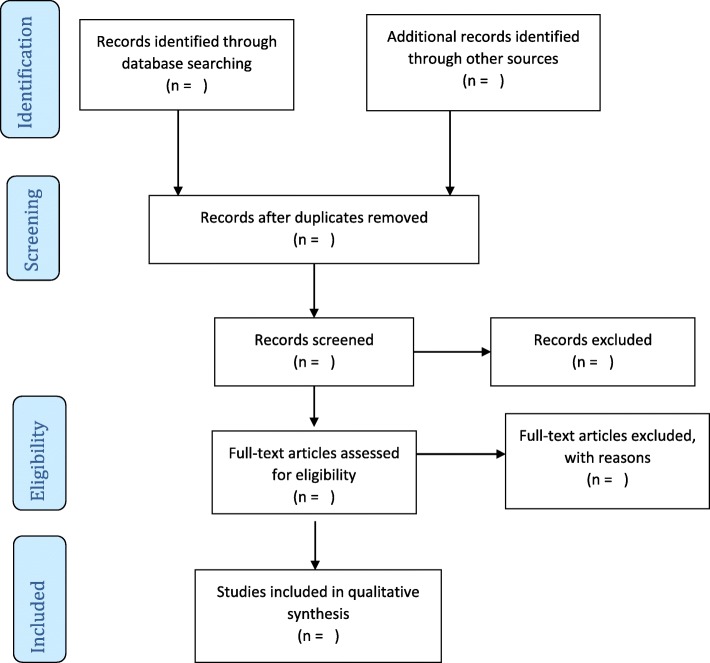
PRISMA 2009 flow diagram

A comprehensive search will be conducted in the databases indicated above using the keyword combinations and imported into Mendeley Desktop. Duplicates will be removed, and the title screening performed by two reviewers in parallel using the eligibility criteria. Abstract and full-text screening of the studies will also be conducted by two independent reviewers guided by the eligibility criteria. We will seek assistance from the Catholic University College of Ghana or the University of KwaZulu-Natal library services or write to authors requesting full-text articles not available online. Disagreements among reviewers following abstract screening will be resolved through discussions to build consensus. However, a third reviewer will be involved to address discrepancies between reviewers at the full-text screening stage. The inter-rater agreement (Cohen’s kappa coefficient (κ) statistic) between reviewers will be calculated following full article screening as well as the McNemar’s chi-square statistic using Strata 14. Details of the search records such as date of search, database, keywords, number of studies identified, and the number of eligible studies will be appropriately documented. The authors will follow an adapted PRISMA (Preferred Reporting Items for Systematic Reviews and Meta-Analysis) guideline to report the screening results [16], as shown in Fig. 1.

### Charting the data

The reviewers will extract information relevant to the aim of the proposed scoping review. A data extraction form will be developed electronically using Google forms and piloted with 10% of the included studies by two investigators to ensure accuracy and consistency of the extracted data [17]. Any feedback from the investigators will be used to update the data extraction form before its final adoption. Table 3 shows the form that will be used to extract data relevant to address the research question.

**Table 3:** Data extraction form

Author and date of publication4	
Study title	
Study aim/objective	
Type of study design	
Study setting (country)	
Geography setting (rural/urban)	
Study population (age and sex)	
Sample size	
Study findings	
Significant findings	
Conclusions	

The data extraction form will include the following information: author with date, study title, study design, country where the study was conducted, population, intervention, outcomes, and other relevant findings.

### Collating, summarizing, and reporting the results

The aim of the proposed scoping review is to map the existing evidence on the burden and distribution of childhood obesity, summarize the results as reported in the included studies, and identify gaps for future research. A narrative approach will be used to report the findings from the included studies through thematic content analysis. NVivo version 11.0 software will be employed to extract the relevant themes. The themes will be collated, summarized, and reported around the following outcomes: prevalence, incidence, mortality, trends, and risk factors of childhood obesity. Emerging themes will also be reported.

### Quality appraisal

The Mixed Method Quality Appraisal Tool (MMAT) Version 2018 [18] will be used to assess the methodological quality of all included studies. The MMAT will be used to examine included studies according to the following categories: the appropriateness of the aim of the study, adequacy and methodology, study design, participant recruitment, data collection, data analysis, and presentation of findings. The quality appraisal will enable the reviewers to report on the risk of bias of the included studies and the overall quality of evidence that will be reported.

## Discussion

This proposed scoping review will map evidence of existing literature on the burden and distribution of childhood obesity in SSA. Children between the ages of 2 and 15 years have been identified to have an increased risk of obesity and its related health problems [3, 4, 6], especially in LMICs [5]. The BMI percentile charts for age and sex have been widely accepted as an appropriate and convenient tool for assessing body composition among children [10, 12]. Other studies have also established linkages between obesity and some socio-demographic factors [19–22]. The researchers, therefore, intend to map literature from 2009 to 2019 to obtain the most recent information on the burden and distribution of childhood obesity in SSA. This study will focus on evidence reported in SSA due to the increasing burden of childhood obesity generally in LMICs [2, 5].

We anticipate that the proposed study will contribute to the existing body of knowledge on childhood obesity, identify gaps in knowledge on the topic, inform future research direction, and provide evidence-based information to strengthen health systems and policies on childhood obesity towards achieving the WHO global target of halting the rise in obesity by 2025 [23].

## Conclusion

The findings of this systematic scoping review will provide evidence that will be beneficial to future research such as systematic reviews, meta-analysis, and primary studies to influence policy and implementation of strategies to end childhood obesity.

## Supplementary information

**Additional file 1.** PRISM-P

## Data Availability

All studies, documents, and reports used for this study are duly cited and presented in the form of references.

## Abbreviations

BMI: Body mass index
LMICs: Low- and middle-income countries
SSA: Sub-Saharan Africa
WHO: World Health Organization
